# Supplementary material for: Longitudinal Characterization of a Neutralizing and Total Antibody Response in Patients with Severe COVID-19 and Fatal Outcomes
Source: Vaccines (Basel). 2022 Dec 1;10(12):2063. doi: 10.3390/vaccines10122063 (PMC9785259; doi:10.3390/vaccines10122063)
Supplement: Supplementary file 1 [file vaccines-10-02063-s001.zip › vaccines-2025134-supplementary.pdf]

Table S1. Statistical differences in Figure 1. Intragroup and intergroup comparisons. An intergroup comparison (global) was made for each follow up, comparing all 3 study groups with a Kruskal Wallis test. Intergroup comparisons between pairs of study groups was made with a Wilcoxon rank sum test.

Comparison	Initial Follow-up	Follow-up 1	Follow-up 2	Follow up 3
Global (Kruskal Wallis)	$p < 0.001$	p = 0.42	$p = 0.87$	$p = 0.1$
Non Hospitalized vs Hospitalized Recovered (Wilcoxon)	$p < 0.001$	p = 0.5	$p = 0.63$	$P = 0.087$
Non Hospitalized vs Hospitalized Deceased (Wilcoxon)	$p < 0.001$	p = 0.24	$p = 0.64$	$p = 0.056$
Hospitalized Recovered vs Hospitalized Deceased (Wilcoxon)	$p = 0.35$	p = 0.96	$p = 0.89$	$p = 0.31$

Table S2. Statistical differences in Figure 2. Intragroup and intergroup comparisons. An intergroup comparison (global) was made for each follow up, comparing all 3 study groups with a Kruskal Wallis test. Intergroup comparisons between pairs of study groups was made with a Wilcoxon rank sum test.

Comparison	Initial Follow-up	Follow-up 1	Follow-up 2	Follow up 3
Global (Kruskal Wallis)	$p < 0.001$	$p = 0.064$	$p = 0.013$	$p = 0.095$
Non Hospitalized vs Hospitalized Recovered (Wilcoxon)	$p < 0.001$	$p = 0.043$	$p = 0.0051$	$p = 0.036$
Non Hospitalized vs Hospitalized Deceased (Wilcoxon)	$p < 0.001$	$p = 0.021$	$p = 0.0067$	$p = 0.064$
Hospitalized Recovered vs Hospitalized Deceased (Wilcoxon)	$p = 0.53$	$p = 0.62$	$p = 0.94$	$p = 0.75$

Table S3. Statistical differences in Figure 3. Intragroup and intergroup comparisons. An intergroup comparison (global) was made for each follow up, comparing all 3 study groups with a Kruskal Wallis test. Intergroup comparisons between pairs of study groups was made with a Wilcoxon rank sum test.

Comparison	Initial Follow-up	Follow-up 1	Follow-up 2	Follow up 3
Global	p = 0.0069	p = 0.0026	p = 0.0015	p = 0.021
Wuhan vs B.1.1.519	p = 0.041	p = 0.97	p = 0.45	p = 0.24
Wuhan vs Delta	p = 0.004	p = 0.022	p = 0.053	p = 0.11
Wuhan vs Omicron	p = 0.018	p = 0.0015	p = 0.0014	p = 0.047
B.1.1.519 vs Delta	p = 0.36	p = 0.048	p = 0.023	p = 0.031
B.1.1.519 vs Omicron	p = 0.13	p = 0.007	p = 0.0028	p = 0.16
Delta vs Omicron	p = 0.46	p = 0.049	p = 0.034	p = .029

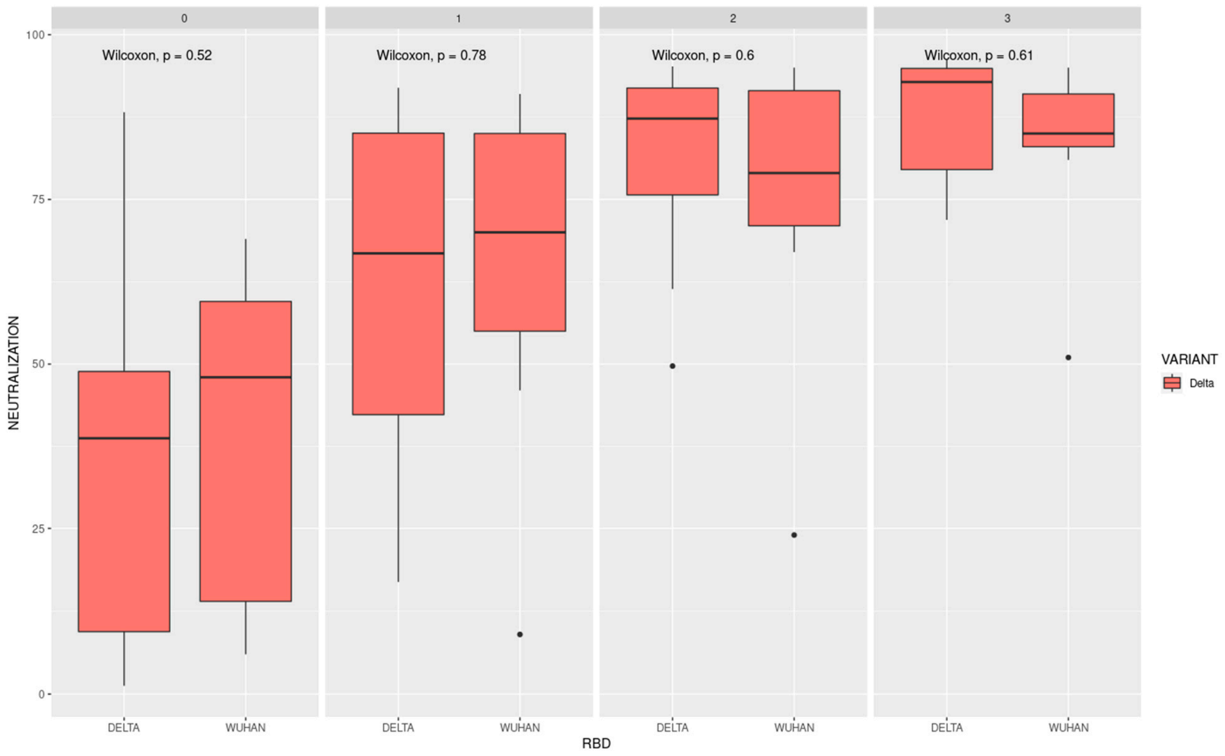

Figure S1. Comparison of neutralization values using Delta RBD substrate. Boxplot of 4 panels, each panel represents one follow up measurement, comparing median neutralization values and IQ ranges, with a coupled wilcoxon test. No statistically significant findings.

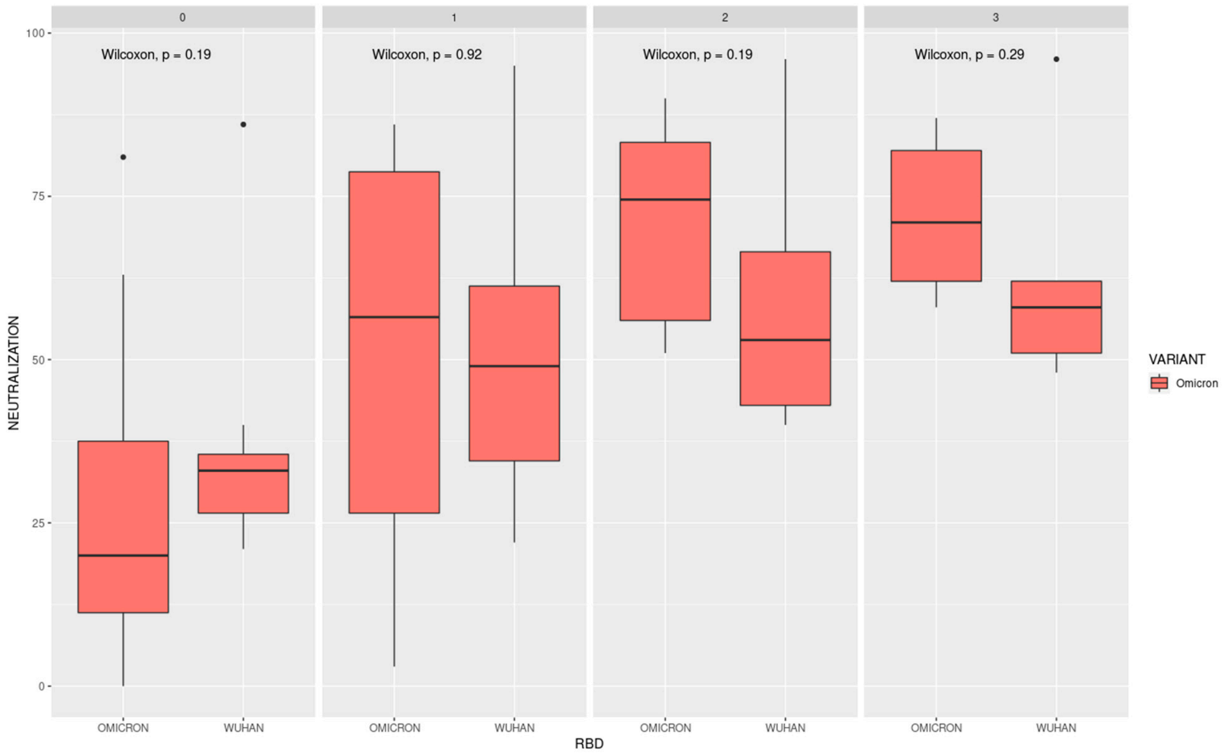

Figure S2. Comparison of neutralization values using Omicron RBD substrate. Boxplot of 4 panels, each panel represents one follow up measurement, comparing median neutralization values and IQ ranges, with a coupled wilcoxon test. No statistical significant findings,
